# Association between the mid-upper arm circumference (MUAC) and calf circumference (CC) screening indicators of sarcopenia with the risk of pneumonia in stable patients diagnosed with schizophrenia

**DOI:** 10.3389/fpsyt.2022.931933

**Published:** 2022-08-26

**Authors:** Silan Ren, Sha Huang, Ming Chen, Tian Zhu, Qiuxia Li, Xiaoyan Chen

**Affiliations:** ^1^Department of Nursing, Sichuan Vocational College of Health and Rehabilitation, Zigong, China; ^2^Zigong Affiliated Hospital of Southwest Medical University, Zigong Psychiatric Research Center, Zigong, China; ^3^Psychiatric Hospital of Ziyang, Ziyang, China

**Keywords:** MUAC, mid-upper arm circumference, CC, calf circumference, schizophrenia, pneumonia

## Abstract

**Aim:**

Here, we investigate the relationship between mid-upper arm circumference (MUAC) and calf circumference (CC) screening indicators of sarcopenia and the risk of pneumonia in stable patients diagnosed with schizophrenia.

**Method:**

The study is prospective and includes inpatients with schizophrenia from two mental health centers in Western China. The studied screening indicators, MUAC and CC were assessed in standing patients. The relationship between MUAC and CC as sarcopenia screening indicators with the risk of pneumonia in patients with schizophrenia was analyzed by performing a statistical logistic regression analysis.

**Result:**

For this study, 339 patients with schizophrenia, aged 50 years and over were recruited. Moreover, four patients with pneumonia that occurred within 1 week of the relapse of schizophrenia were excluded. As a result, only 335 patients were included in the analysis. Pneumonia has been reported in 82 (24.5%) of all included patients with schizophrenia. Our data analysis confirmed that in the male patients, the higher CC was associated with a lower risk of pneumonia (odds ratio [OR] = 0.751, 95% *CI*: 0.635–0.889). We have divided men into two cohorts following the values of CC. Our analysis further showed that the patients with CC ≥ 34 cm had a lower risk of pneumonia in men (*OR* = 0.36, 95% *CI*: 0.163–0.795).

**Conclusion:**

We demonstrate that CC is associated with pneumonia risk in stable men with schizophrenia.

## Introduction

Sarcopenia, also called muscle atrophy, is an aging-associated muscle debilitation and decline in the overall body performance with age (1). Data show that the increased risk of pneumonia is associated with sarcopenia in patients with alcoholic hepatitis, esophageal cancer surgery, colorectal cancer surgery, and older age (2–5).

People's prejudice against patients with schizophrenia, so the social acceptance is low, it is not easy to return to society (6), and schizophrenia has a genetic susceptibility (7). Some families also have mental patients, there is no extra staff and financial care for patients with schizophrenia, and it is difficult to return to the family. Therefore, many patients with schizophrenia can only stay in the ward even after they recover. People with schizophrenia live in a relatively closed environment for a long time, have reduced physical activity, and have a relatively simple diet, which may lead to an increased risk of sarcopenia (8). Interestingly, some authors reported the presence of a similar cytokine (Interleukin-6 [IL-6]) in both patients with sarcopenia and schizophrenia (9). In addition, pneumonia was reported to be significantly higher in patients with schizophrenia than in the general population (10). Combining the above studies, we consider that pneumonia in schizophrenia may be associated with sarcopenia. Therefore, the detection of sarcopenia in patients with schizophrenia is very important in the course of its treatment.

In clinical practice, decreased muscle mass is diagnosed by magnetic resonance imaging (MRI), computed tomography (CT), dual-energy X-ray absorptiometry (DXA), and bioelectrical impedance analysis (BIA) (1). Some of these methods are expensive, some are radioactive, and some are interfering with food intake and the general patient's health status (11). Therefore, the search for inexpensive, safe, and food- and general-health status independent methods for early sarcopenia diagnosis to predict pneumonia is of great interest. Studies have found that mid-upper arm circumference (MUAC) and calf circumference (CC) can be used as simple proxy screening for sarcopenia (12–14).

However, the relationship between MUAC and CC (as surrogate indicators for sarcopenia) and the risk of pneumonia in stable patients with schizophrenia was not yet well-established. Therefore, we explore the association among MUAC, CC, and pneumonia in stable schizophrenia. We hypothesized that the lower values of patients' MUAC and CC indicators were linked with an increased risk of pneumonia in stable schizophrenia.

## Methods

### Study design and patients' characteristics

Inpatients diagnosed with stable schizophrenia from two mental health centers in Western China were enrolled in the study and established the cohort of sarcopenia. Baseline patients' data were collected from 1 September to 30 September 2020. Additionally, we gathered clinical data from patients with pneumonia diagnosed and treated at two mental health centers between October 2020 and October 2021. Our baseline inclusion and exclusion criteria for this study were based on a previous article (14). At the same time, during our follow-up period, if pneumonia occurred within 1 week after the change of condition, we excluded it.

The study was performed following the Declaration of Helsinki and was legitimated by the Institutional Review Board (IRB) of the two mental health centers in Western China (IRB number: 20191001; zjsjsbyy-kyxm-2019-2). All patients or their legitimate custodians signed an informed consent form.

### Anthropometric measurements

Both MUAC and CC as screening indicators of sarcopenia were measured while the patients were upright. At this point, tape measures at the midpoint between the lateral projection of the scapula acromion and the lower border of the olecranon were performed with the arm flexed at 90 degrees. To measure CC, we measured the maximum circumference of the lower leg. Both indicators were calculated to the closest decimal point in centimeters. The two measurements on the leading lateral were averaged. All measurements were performed by trained professionals from the medical centers where the study was executed. The patients were divided into 2 groups according to the CC cut-off value recommended by the Asian Working Group for Sarcopenia (AWGS) 2019 diagnostic consensus, and sarcopenia was considered to be less than 34 cm in men and 33 cm in women (1). Since MUAC has no recommended cut-off value for the consensus, no grouping was performed (1).

### Outcome indicator

Pneumonia was diagnosed by a clinician and was treated with drugs, mainly antibiotics. In the current study, only patients who developed pneumonia for the first time during follow-up were enrolled. Each patient had a chest radiograph or/and CT scan. Pneumonia was defined as an acute infection of the lung parenchyma, associated with acute infiltrates on chest radiograph or CT, accompanied by the prevalence of two or more symptoms, such as increased body temperature (≥38°C), hypothermia (<36°C), chills, sweating, novel cough, or respiratory tract change in color of discharge, chest discomfort, or difficulty in breathing (15).

### Covariates

Covariate data were collected from patients diagnosed with schizophrenia who had been hospitalized for more than 6 months in the hospital through an electronic system to find out the corresponding ward area. After that, their basic medical data were collected face-to-face according to the inclusion criteria. We collected the baseline data of each patient, including age, sex, weight, height, smoking, and drinking history. All reported chronic illnesses, such as diabetes, increased blood pressure, coronary heart disease (CHD), chronic obstructive pulmonary disease (COPD), and hyperlipidemia were also recorded and were assessed as covariates in the analysis. In addition, the Activities of Daily Living (ADL) scores, based on the ADL assessment (16), Patient Health Questionnaire-9 (PHQ-9) depression scores, based on the PHQ-9 (17), Short Portable Mental Status Questionnaire (SPMSQ) cognitive function marks, based on the SPMSQ measurement (18), frail score (19), and antipsychotic (typical antipsychotic and drug kinds), and benzhexol as covariates were included in the study. Diabetes, hypertension, CHD, COPD, and hyperlipidemia as comorbidities met the respective diagnostic criteria (20). In this study, a single factor <0.05 was included in the model correction. We constructed two models: Model 1: a non-adjusted model and Model 2: adjusted for covariates, such as COPD, PHQ-9, and Body-Mass Index (BMI) in men and COPD and BMI in women. Although there were differences in the distribution of smoking history among women, the frequency of occurrence was too small to be corrected for inclusion.

### Statistical analyses

The SPSS 25.0 software was applied for the statistical analyses of all gathered patients' data. The values of *p* less than 0.05 were assigned as the threshold for significance and all tests were two-sided. The ordinarily dispersed results were represented as mean ± standard deviation (SD), otherwise, the median (quartile) was provided. The Rank-sum test, Student's *t*-test, and Pearson's chi-square test were employed for the comparison of baseline features. The logistic regression analysis determined the relationship between MUAC and CC values with the risk of pneumonia in patients with schizophrenia.

## Results

**Figure 1:**
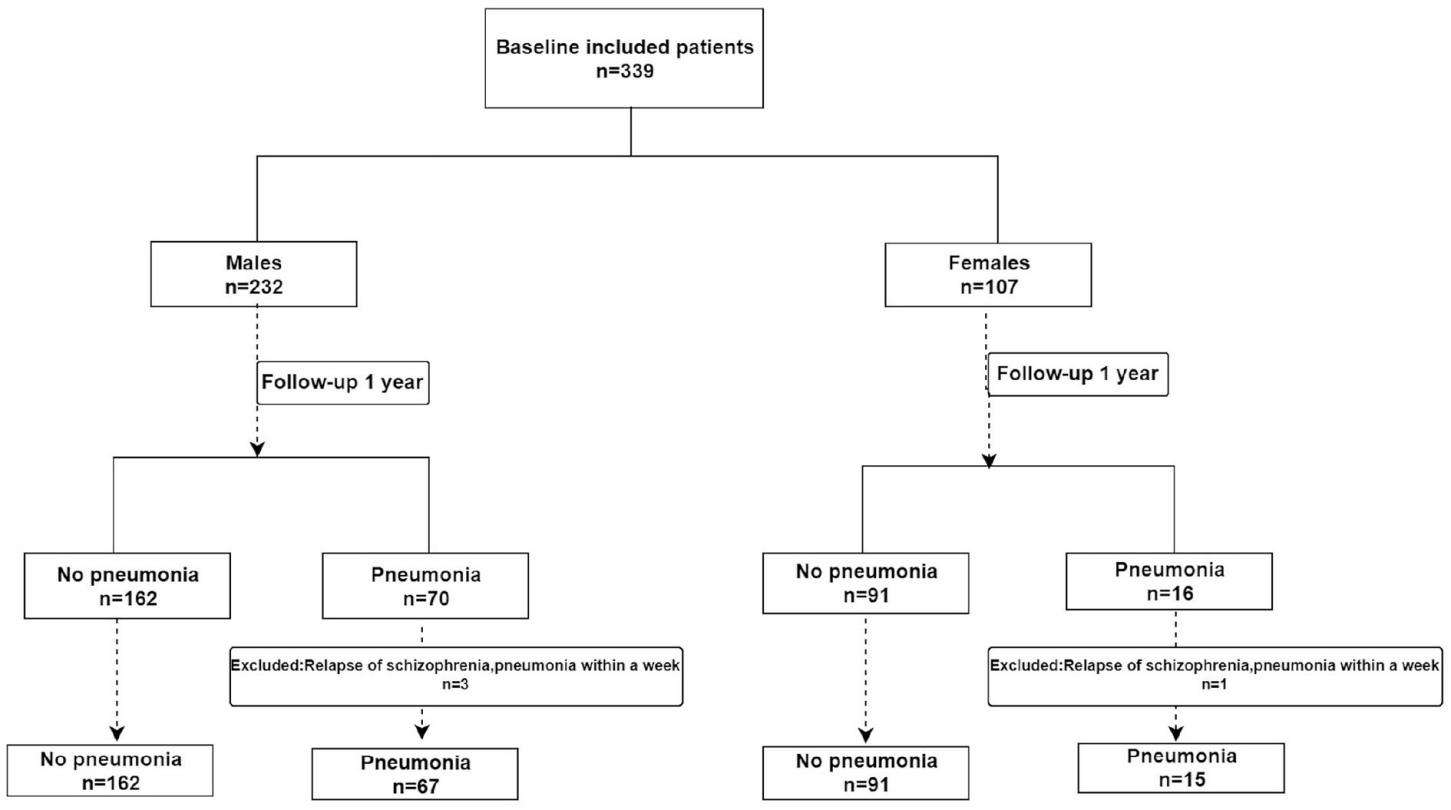
The study profile included patient selection and pneumonia information.

The patients with schizophrenia enrolled in the study included 232 men and 107 women aged 50 years and over. During the follow-up, four patients with pneumonia that occurred within 1 week of the relapse of schizophrenia were excluded. As a result, only 335 patients were included in the analysis (Figure 1). Pneumonia has been reported in 82 (24.5%) of the included schizophrenic patients. In the male patients with schizophrenia, the differences in COPD, PHQ-9 scores, and BMI between pneumonia and non-pneumonia groups were statistically significant. In the female patients, the smoking history, COPD, and BMI were statistically significant among patients with pneumonia and non-pneumonia (Table 1).

**Table 1:** Characteristics of the participants.

**General characteristics**	**Male**			**Female**		
	**Non-pneumonia**	**Pneumonia**	**P**	**Non-pneumonia**	**Pneumonia**	**P**
	***n =*** **162**	***n =*** **67**		***n =*** **91**	***n =*** **15**	
Age, year, median (iqr)	65 (58, 69)	65 (59, 70)	0.307	65 (60,69)	66 (61,73)	0.131
Smoking history, *n* (%)			0.573			0.013
No	96 (72.18)	37 (27.82)		91 (86.67)	14 (13.33)	
Yes	66 (68.75)	30 (31.25)		0	1 (100%)	
Drinking history, *n* (%)			0.653			-
No	130 (71.43)	52 (28.57)		91 (85.85)	15 (14.15)	
Yes	32 (68.09)	15 (31.91)		0	0	
COPD, *n* (%)			0.001			0.003
No	132 (76.74)	40 (23.26)		89 (88.12)	12 (11.88)	
Yes	30 (52.63)	27 (47.37)		2 (40)	3 (60)	
Hypertension, *n* (%)			0.451			0.451
No	126 (72)	49 (28)		69 (87.34)	10 (12.66)	
Yes	36 (66.67)	18 (33.33)		2 2 (81.48)	5 (18.52)	
Diabetes, *n* (%)			0.914			0.106
No	132 (70.59)	55 (29.41)		72 (88.89)	9 (11.11)	
Yes	30 (71.43)	12 (28.57)		19 (76)	6 (24)	
CHD, *n* (%)			0.645			0.089
No	158 (70.54)	66 (29.46)		88 (87.13)	13 (12.87)	
Yes	4 (80)	1 (20)		3 (60)	2 (40)	
Hyperlipidemia, *n* (%)			0.749			0.36
No	145 (71.08)	59 (28.92)		85 (86.73)	13 (13.27)	
Yes	17 (68)	8 (32)		6 (75)	2 (25)	
ADL score, *n* (%)			0.165			0.441
100 score	103 (74.1)	36 (25.9)		40 (88.89)	5 (11.11)	
<100 score	59 (65.56)	31 (34.44)		51 (83.61)	10 (16.39)	
PHQ-9 score, *n* (%)			0.002			0.441
≤ 4	121 (77.07)	36 (22.93)		58 (87.88)	8 (12.12)	
>4	41 (56.94)	31 (43.06)		33 (82.5)	7 (17.5)	
SPMSQ score, *n* (%)			0.41			0.315
0–2	55 (74.32)	19 (25.68)		30 (90.91)	3 (9.09)	
3–10	107 (69.03)	48 (30.97)		61 (83.56)	12 (16.44)	
BMI, kg/m^2^, mean (SD)	24.69 (3.93)	23.5 (3.54)	0.032	24.21 (4.14)	21.58 (5.03)	0.029
Typical antipsychotic, *n* (%)			0.968			0.352
No	157 (70.72)	65 (29.28)		86 (85.15)	15 (14.85)	
Yes	5 (71.43)	2 (28.57)		5 (100)	0	
Antipsychotic, *n* (%)			0.166			0.692
Alone	140 (72.54)	53 (27.46)		82 (85.42)	14 (14.58)	
Combine	22 (61.11)	14 (38.89)		9 (90)	1 (10)	
Benzhexol, *n* (%)			0.116			0.144
No	117 (68.02)	55 (31.98)		62 (82.67)	13 (17.33)	
Yes	45 (78.95)	12 (21.05)		29 (93.55)	2 (6.45)	
Frail score, median (iqr)	1 (1, 2)	1 (1, 2)	0.204	1 (0, 2)	1 (1, 3)	0.111

COPD, chronic obstructive pulmonary disease; CHD, coronary heart disease; ADL, activities of daily living; PHQ, Patient Health Questionnaire; SPMSQ, Short Portable Mental Status Questionnaire; BMI, body-mass index. The Rank-sum test, Student's *t* test and Pearson's chi-square test were employed for comparison of baseline features.

**Figure 2:**
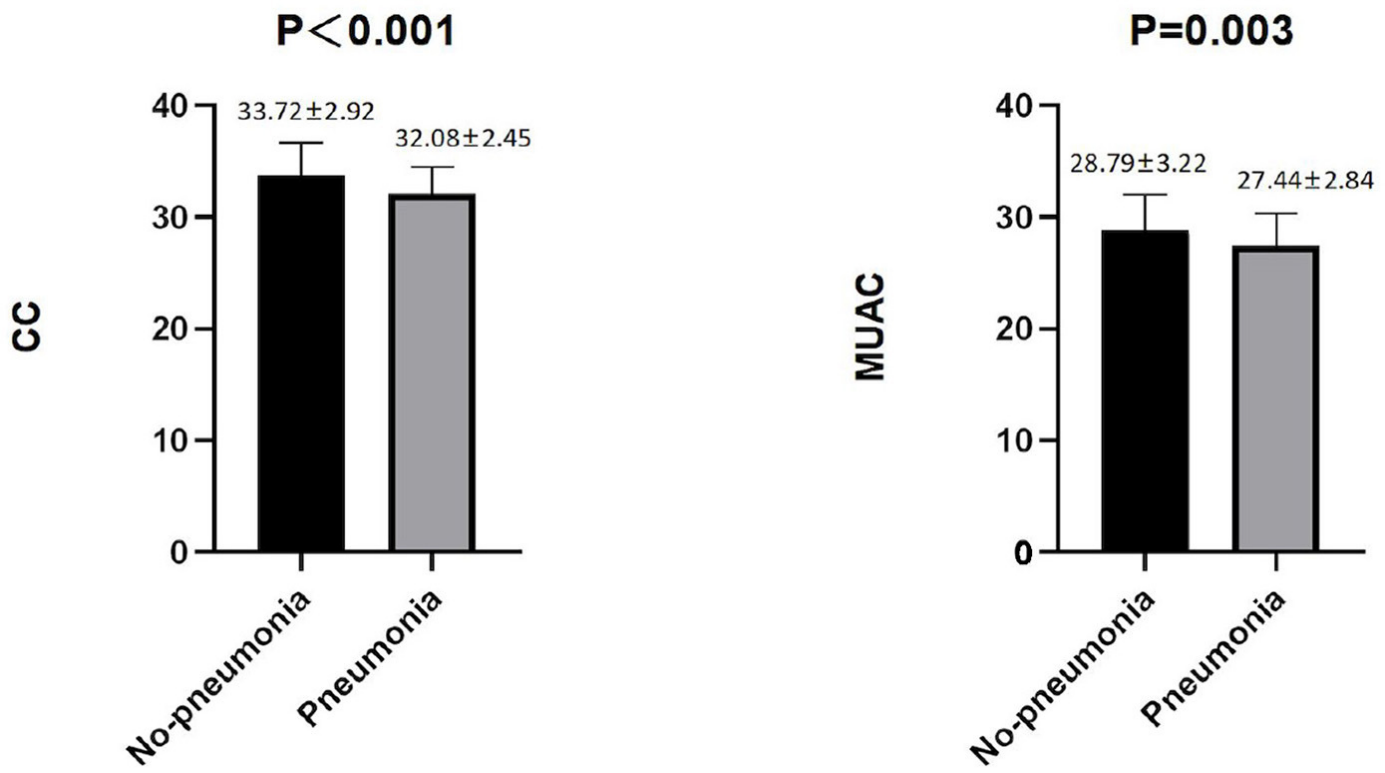
Differences in CC and MUAC between pneumonia and non-pneumonia in males. The Student's *t-*test was employed for comparison of two groups.

**Figure 3:**
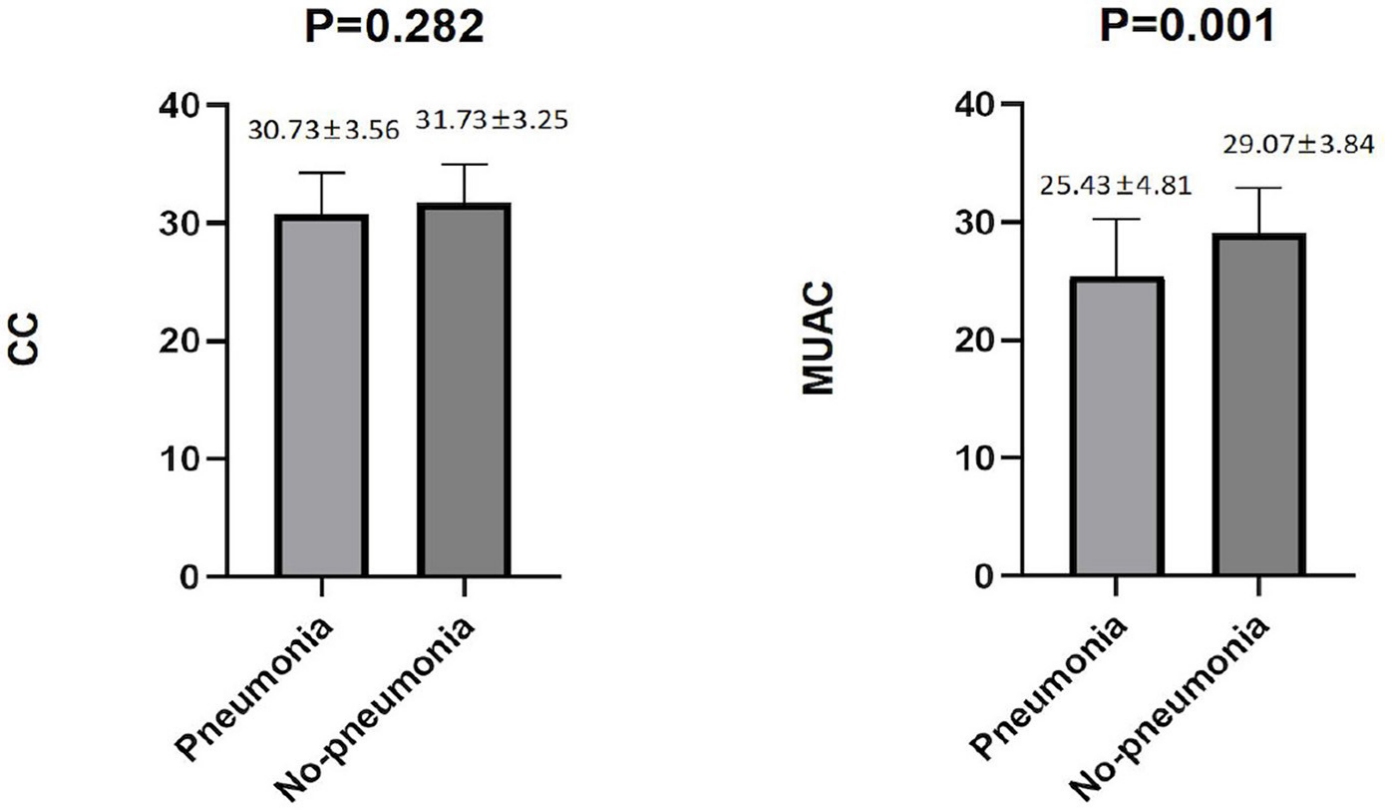
Differences in CC and MUAC between pneumonia and non-pneumonia in females. The Student's *t*-test was employed for comparison of two groups.

Among the male patients with schizophrenia, those with pneumonia had lower CC and MUAC measurements when compared with the non-pneumonia ones. The statistical analysis of the measured CC values showed *p* < 0.001, while for MUAC values *p* was equal to 0.003 (Figure 2). When all male patients with schizophrenia were divided into two categories according to the values of CC (34 cm), the detected prevalence of pneumonia was higher among those with CC <34 cm than with CC ≥ 34 cm (37.59% vs. 17.71%, *p* = 0.001). These data are presented in Table 2. Among the female patients with schizophrenia, the MUAC measurements in the patients with pneumonia were smaller than that of non-pneumonia ones (*p* = 0.001, Figure 3). However, the differences in the measured CC values in pneumonia and non-pneumonia female patients with schizophrenia were not statistically significant (Figure 3 and Table 2).

**Table 2:** Univariate analysis of pneumonia and CC.

**Variable**	**Male (*****n*** **= 229)**			**Female (*****n*** **= 106)**		
	**No pneumonia**	**Pneumonia**	***P***	**No pneumonia**	**Pneumonia**	***P***
	**(*n =* 162)**	**(*n =* 67)**		**(*n =* 91)**	**(*n =* 15)**	
			0.001			0.974
CC ≥ 34 (M) or CC ≥ 33 (F), cm, *n* (%)	79 (82.29)	17 (17.71)		36 (85.71)	6 (14.29)	
CC < 34 (M) or CC < 33 (F), cm, *n* (%)	83 (62.41)	50 (37.59)		55 (85.94)	9 (14.06)	

M, male; F, female.

We found that in male patients, higher CC and MUAC scores in patients with schizophrenia were associated with a lower risk of pneumonia (CC: odds ratio [*OR*] = 0.805, 95% *CI*: 0.719–0.901; MUAC: *OR* = 0.867, 95% *CI*: 0.787–0.956; Table 3). After adjusting for some confounding factors, CC remained a protective factor for pneumonia (*OR* = 0.751, 95% *CI*: 0.635–0.889; Table 3). We further divided all male patients with schizophrenia into two categories taking into account 34 cm as the cut-off value for CC and found that patients with CC ≥ 34 cm had a lower risk of pneumonia (*OR* = 0.357, 95% *CI*: 0.19–0.671; Table 3). We adjusted the model for certain confounding factors, and the risk of pneumonia appeared reduced in individuals with CC ≥ 34 cm (*OR* = 0.36, 95% *CI*: 0.163–0.795; Table 3). Among women with schizophrenia, higher MUAC was associated with a lower risk of pneumonia (*OR* = 0.8, 95% *CI*: 0.69–0.928; Table 3). After adjusting for certain confounders, we found that MUAC was not associated with higher pneumonia risk (Table 3).

**Table 3:** Correlations between CC, MUAC and pneumonia.

**Variable**	**Model 1**		**Model 2**	
	***P*** **-value**	**OR (95% *CI*)**	***P*** **-value**	**OR (95% *CI*)**
Male				
CC^*^	< 0.001	0.805 (0.719–0.901)	0.001	0.751 (0.635–0.889)
CC < 34 cm	-	1	-	1
CC ≥ 34 cm	0.001	0.357 (0.19–0.671)	0.011	0.36 (0.163–0.795)
MUAC^*^	0.004	0.867 (0.787–0.956)	0.08	0.865 (0.735–1.018)
Female				
CC^*^	0.281	0.909 (0.765–1.081)	0.626	1.063 (0.831–1.36)
CC <33 cm	-	1	-	1
CC≥33 cm	0.974	1.019 (0.334–3.107)	0.054	5.002 (0.97–25.804)
MUAC^*^	0.003	0.8 (0.69–0.928)	0.051	0.82 (0.673–1.001)

* : continuous variable. Model 1: a non-adjusted model. Model 2: adjusting for COPD, PHQ-9, BMI in males; adjusting for COPD, BMI in females.

## Discussion

Our study found that higher CC was associated with a lower risk of pneumonia in men with schizophrenia. To our knowledge, this investigation is the first to explore the relationship between two common sarcopenia screening tools, MUAC and CC, and the risk of pneumonia in patients with stable schizophrenia. The study further enables clinicians to assess the risk of pneumonia in stable male patients with schizophrenia with simple and reproducible anthropometric measures. It further enhances dynamic assessment during disease intervention. This is an advantage of the study as in clinical practice, the benefits of introducing CC measurements come from their simplicity and rapidity, as well as their economical and non-invasive nature.

In our study, CC, one of the screening markers for sarcopenia, was associated with pneumonia risk in patients with schizophrenia. People with sarcopenia have reduced interleukin-15, which has an effect on the immune system, especially on natural killer cells (21). In addition, the phosphoinositide 3-kinase (PI3K)-Akt pathway in patients with sarcopenia is also affected, thereby affecting part of the function of neutrophils (22). On the other hand, people with sarcopenia have reduced muscle strength throughout the body, including respiratory muscles, which is one of the risk factors for pneumonia because they are unable to cough effectively to clear their airways (23). Finally, patients with low muscle mass generally have poorer oral hygiene (24). The study by Juthani-Mehta et al. showed that impaired oral hygiene is one of the risk factors for pneumonia (25). That may explain the increased risk of pneumonia in patients with sarcopenia. But our results were meaningful only in men, not women. We believe that this may be due to higher leg fat mass in women than in men (26) and therefore, the association between CC and muscle mass in women is not as strong as in men (27). Additionally, Weiss et al. found a different degree of association between muscle sonography and CC between the two sexes (0.8 in men and 0.76 in women) (28).

MUAC is recommended as a screening marker for the nutritional status and sarcopenia, which is associated with muscle mass and subcutaneous fat, is less affected by fluid retention, and is easy to perform at no additional cost (13, 14). However, in our study, MUAC was not associated with the risk of pneumonia, and the same results were obtained in the study by Yardimci et al. (29). We speculate that it may be related to less muscle mass in the upper limbs than in the lower limbs. Ma et al. found that MUAC is one of the independent predictors of in-hospital mortality in older patients with pneumonia (30). However, our study has a short follow-up time and collected fewer death data, and it is not yet possible to confirm whether MUAC is associated with death in patients with stable schizophrenia. There is currently an ongoing follow-up.

The incidence of pneumonia in patients with schizophrenia was 24.5% in our study. Chou et al. conducted a 9-year follow-up study in Taiwan and found that 10.26% of patients with schizophrenia developed pneumonia (31). The mean age of the patients in their study was 40.62 years, while the patients in our study were all 50 years of age and older. We guess that the reason for the higher incidence of pneumonia in our study may be the advanced patients' age under our investigation.

The current study has certain limitations too. First, we did not collect information on the severity of pneumonia and there is currently little follow-up information on the patients with death data to further investigate the association of MUAC and CC with pneumonia severity and death. However, the follow-up studies continue and this will address this issue in the future. Second, the cohort of this study was small, including only a small number of patients with schizophrenia in Western China, so the results cannot be generalized to other races and countries, and validation with a larger cohort and a larger population is strongly needed. Third, we did not perform a categorical analysis of MUAC due to the lack of recommended cut-off values for MUAC in current guidelines. Therefore, a recommendation for further research is generally a huge need.

## Conclusion

Our study has demonstrated that lower CC scores were associated with increased pneumonia risk in stable patients with schizophrenia in men, but not in women. Therefore, we recommend that in men with stable schizophrenia, CC can be applied as a solid screening marker for sarcopenia to identify patients who may be at risk for pneumonia.

## Data availability statement

The raw data supporting the conclusions of this article will be made available by the authors, without undue reservation.

## Ethics statement

The studies involving human participants were reviewed and approved by Institutional Review Board of the Zigong Medical Foundation (IRB number: 20191001) and the Psychiatric Hospital of Ziyang (IRB number: zjsjsbyy-kyxm-2019-2). The patients/participants provided their written informed consent to participate in this study.

## Author contributions

SR, SH, MC, TZ, QL, and XC: study concept and design. MC, TZ, and QL: acquisition of data. SR, SH, and XC: analysis and interpretation of data and drafting of the manuscript. XC: critical revision of the manuscript for important intellectual content. All authors of this manuscript have fully contributed to the manuscript and approved the final manuscript.

## Funding

This work was funded by the Psychiatric Hospital of Ziyang Support Project (Project No. Zysjsbyy-kyxm-2022-6) and the 2021 Key Science and Technology Plan of Zigong City (Project No. 2021YXY12). The sponsors did not participate in the design, methods, data collection, analysis, or preparation of this manuscript.

## Conflict of interest

The authors declare that the research was conducted in the absence of any commercial or financial relationships that could be construed as a potential conflict of interest.

## Publisher's note

All claims expressed in this article are solely those of the authors and do not necessarily represent those of their affiliated organizations, or those of the publisher, the editors and the reviewers. Any product that may be evaluated in this article, or claim that may be made by its manufacturer, is not guaranteed or endorsed by the publisher.
